# Cognitive and Clinical Dysfunction, Altered MEG Resting-State Networks and Thalamic Atrophy in Multiple Sclerosis

**DOI:** 10.1371/journal.pone.0069318

**Published:** 2013-07-31

**Authors:** Prejaas Tewarie, Menno M. Schoonheim, Cornelis J. Stam, Marieke L. van der Meer, Bob W. van Dijk, Frederik Barkhof, Chris H. Polman, Arjan Hillebrand

**Affiliations:** 1 Department of Neurology, VU University Medical Center, Amsterdam, The Netherlands; 2 Department of Clinical Neurophysiology and Magnetoencephalography Center, VU University Medical Center, Amsterdam, The Netherlands; 3 Department of Radiology and Nuclear Medicine, VU University Medical Center, Amsterdam, The Netherlands; 4 Department of Anatomy & Neuroscience, VU University Medical Center, Amsterdam, The Netherlands; 5 Department of Physics and Medical Technology, VU University Medical Center, Amsterdam, The Netherlands; University College of London - Institute of Neurology, United Kingdom

## Abstract

The relation between pathological findings and clinical and cognitive decline in Multiple Sclerosis remains unclear. Here, we tested the hypothesis that altered functional connectivity could provide a missing link between structural findings, such as thalamic atrophy and white matter lesion load, and clinical and cognitive dysfunction. Resting-state magnetoencephalography recordings from 21 MS patients and 17 gender- and age matched controls were projected onto atlas-based regions-of–interest using beamforming. Average functional connectivity was computed for each ROI and literature-based resting-state networks using the phase-lag index. Structural measures of whole brain and thalamic atrophy and lesion load were estimated from MRI scans. Global analyses showed lower functional connectivity in the alpha2 band and higher functional connectivity in the beta band in patients with Multiple Sclerosis. Additionally, alpha2 band functional connectivity was lower for the patients in two resting-state networks, namely the default mode network and the visual network. Higher beta band functional connectivity was found in the default mode network and in the temporo-parietal network. Lower alpha2 band functional connectivity in the visual network was related to lower thalamic volumes. Beta band functional connectivity correlated positively with disability scores, most prominently in the default mode network, and correlated negatively with cognitive performance in this network. These findings illustrate the relationship between thalamic atrophy, altered functional connectivity and clinical and cognitive dysfunction in MS, which could serve as a bridge to understand how neurodegeneration is associated with altered functional connectivity and subsequently clinical and cognitive decline.

## Introduction

Multiple Sclerosis (MS) is an inflammatory demyelinating and neurodegenerative disease leading to clinical and cognitive decline to varying degree. Despite much progress in the MS field in recent years, the relation between pathological findings and clinical and cognitive decline remains unclear. The discrepancy between classical MRI findings such as white matter lesion load on the one hand and clinical decline on the other hand has been called the clinico-radiological paradox [1]. To overcome this paradox the emphasis of research has shifted from white matter pathology to gray matter pathology [2]. Gray matter pathology such as atrophy and cortical lesions seem to correlate better with physical disability and cognitive impairment than white matter pathology [3], [4]. Thalamic atrophy in particular seems to be present even in the earlier phases of the disease and is a strong predictor of cognitive decline [3]–[7].

Other insights that may help to solve the clinico-radiological paradox may be gained by studying functional connectivity (FC). Functional MRI (fMRI) studies have revealed a link between altered FC and cognitive symptoms in MS [8]–[12]. Both higher and lower FC has been observed in MS, where higher FC is often interpreted as a “compensatory mechanism” for structural deficits [8], [12]. Alternatively, an higher FC could also be maladaptive and contribute to cognitive worsening [11], or excessive FC could simply be due to pathological disinhibition.

Additional insights may be gained by estimating FC in resting-state networks (RSNs). These RSNs are semi-independent networks that can be captured from the brain's activity during rest and in the absence of goal directed neural activity [13]. One of these networks, the default mode network, may be of special interest here since seems to play a crucial role in cognitive decline in several disorders [14], [15], including MS [11], [16], [17]. In MS, higher FC has also been related to physical disability in other resting-state networks (RSNs), such as the frontoparietal network and the prefronto-insular network [18].

Although fMRI is a powerful tool, its application has some drawbacks. These include its low temporal resolution and, despite progress in understanding the mechanisms that lead to the BOLD (Blood oxygen level dependent) signal, a not-entirely-understood relation with the underlying electrophysiology [19], [20]. Magnetoencephalography (MEG) and Electroencephalography (EEG) are direct measures of neuronal activity with high temporal resolution and thus provide complementary information to fMRI studies [20], and also allow for the investigation of specific RSNs [21]. Task activation MEG studies in MS have revealed lower FC, which has led to the interpretation of MS as a disconnection syndrome [22], [23]. A relatively small resting-state MEG study found lower interhemispheric FC in the alpha band in MS patients with mild disability [24]. The same group partly reproduced these findings but now higher FC in the theta, alpha1, and beta band was found as well [25]. Another study characterised the topology of the functional network as a whole, using a measure that quantifies the “hub-status” of an area (eigenvector centrality, or EC) [26]. Using MEG, lower EC over temporal regions in the alpha2 and beta band and higher EC in the theta band over parietal regions were found. Analyses in these studies were performed at the sensor-level, which are difficult to interpret and may suffer from problems with FC estimation due to field spread [27], [28]. Even approaches where sensor data is projected to source- space may suffer from widespread correlations between reconstructed source elements, leading to similar problems of overestimated FC [29], [30]. An atlas-based MEG beamformer approach in combination with an FC metric that is insensitive to the effects of field spread and volume conduction, i.e. the phase lag index (PLI), has the ability to overcome these problems [27], [31].

Here we used the atlas-based beamformer approach to investigate resting-state MEG FC in the whole brain and in literature based resting-state networks in relation to clinical and cognitive status, and in relation to whole brain- and thalamic atrophy.

## Methods

### General study design

In this cross-sectional study, two independent groups, MS patients and healthy controls, underwent MEG, MRI, neurological examination and neuropsychological assessment on the same day. As outcome measures we used FC estimated from resting-state MEG data, whole brain-, gray matter- and thalamic volumes, clinical status and cognition. FC was assessed between atlas-based ROIs and in literature-based resting-state networks.

### Participants

All subjects from a previous study were included [25]: 34 MS patients (mean age 41.4±8.0 years, disease duration since the first symptom 8.1±1.6 years) and 28 age- and gender matched controls (mean age 39.8±10.5 years). In the present study a number of subjects were excluded due to various reasons: no MRI available (n = 2), too many artefacts and noise in the raw MEG data (see below) (n = 12) and inaccurate MEG/MRI co-registration (n = 10). Consequently, 21 MS patients (mean age 41.9±7.7, disease duration 6.8±0.9 years) and 17 controls (mean age 39.8±9.8) remained in the present study which were still gender- and age matched. All patients were diagnosed with clinically-definite multiple sclerosis [32], specifically the relapsing remitting form of MS (RRMS) [33]. Patients were recruited from the VU University Medical Center. All patients were part of the six-year follow-up of an early inception cohort, in which patients were included at diagnosis and subsequently followed annually [7]. Physical disability was measured using the Expanded Disability Status Scale (EDSS) [34] and found to be relatively mild (median 2, range 0–4.5). Eight patients received interferon treatment since diagnosis. One of these patients switched to glatimere acetate and two to natalizumab which they were receiving during the study. The study protocol was approved by the Local Research Ethics Committee (Medical Ethical Review Committee of VU University Medical Center), whose ethics review criteria conformed to the Helsinki declaration. All subjects had given written informed consent prior to participation.

All subjects underwent a set of neuropsychological tests as described earlier [25]. In summary, the brief repeatable battery for neurological disease (BRB-N), consisting of the selective reminding test (SRT), the 10/36 spatial recall test (SPART), the symbol digit modalities test (SDMT), the word list generation test (WLG), the concept shifting test (CST), the stroop color-word test and the memory comparison test (MCT) were administered. Z-scores were summarized into seven cognitive domains: executive functioning (CST, WLG), verbal memory (SRT), information processing speed (SDMT), visuospatial memory (SPART), working memory (MCT), attention (Stroop) and psychomotor speed (CST, SDMT). In addition a Z–score for overall cognition was calculated. This was overall cognition was calculated by averaging Z-scores of all seven cognitive domains and was used in further analyses [7].

### Magnetic Resonance Imaging

An MRI scan was obtained from all subjects, using a 3T-MRI system (GE Signa HDXT V15m). A 2D dual-echo T2-weighted sequence (TR 9680 ms, TE 22/112 ms) and T1-weighted sequence (TR 475 ms, TE 9 ms) were obtained with 48 slices of 3 mm and 3D-T1 heavily T1-weighted sequence (FSPGR , TR 7.8 ms, TE 3.0 ms, TI 450 ms) with 1 mm, slices covering the entire brain. All scans were inspected by an experienced rater (MMS). Subsequently, lesion volumes were quantified. All lesion volumetric analyses were performed using Alice (Perceptive informatics Inc.) applying a local thresholding technique. Total gray matter (NGMV), total white matter (NWMV), and whole brain volumes (NBV), corrected for head size, were measured using the FSPGR images and SIENAX [35] version 2.5 (part of FSL 4.1, FMRIB's Software Library, http://www.fmrib.ox.ac.uk/fsl). Thalamic volumes were measured using FIRST (part of FSL), providing left and right volumes for the thalamus and were corrected for head size. Left and right thalamic volumes were summed to give the total thalamic volume.

### Magnetoencephalography

MEG data were recorded using a 151-channel whole-head MEG system (CTF systems; Port Coquitlam, BC, Canada) while participants were in a supine position in a magnetically shielded room (Vacuumschmelze, Hanau, Germany). A third-order software gradient [7], [36] was used with a recording passband of 0–150 Hz and a sample frequency of 625 Hz. Participants had to be free of any metal objects. Magnetic fields were recorded during a no-task, eyes-open condition for three minutes and eyes-closed condition for five consecutive minutes. At the beginning and end of each recording, the head position relative to the coordinate system of the helmet was determined by leading small alternating currents through three head position coils attached to the left and right pre-auricular points and the nasion. Changes in head position of <0.5 cm during a recording condition were accepted. Each original dataset consisted of a continuous resting-state recording from which artefact-free segments were subsequently selected for further analysis. Each segment or epoch had a duration of 6.555 seconds (total no. of segments or epochs was 45). Channels and epochs were visually inspected. Epochs and channels were rejected based on system related artefacts (SQUID jumps, noisy, broken or saturated channels), physiological artefacts (eye movements, eye blinks, muscle artefacts), external artefacts (magnetized dental fillings) and environmental noise (PT, AH) [37], as well as for representing an alert eyes-closed state. A minimum of 25 eyes-closed epochs was considered sufficient [38] for further beamformer analysis. On average 5.7 channels (range: 2–14) and 8.4 epochs (range 3–20) were discarded.

### Beamforming: time-series estimation for regions-of-interest

The technique used in this study was recently described [27]. A brief overview is given below. First, a subject's MRI was co-registered with the MEG data through identification of the same anatomical landmarks in the MRI that were also used for the placement of the MEG head-localization coils (i.e. left and right pre-auriculars and nasion). Only data from subjects where the estimated co-registration error was smaller than 0.8 cm were accepted for further analysis. The co-registered MRI was then spatially non-linearly registered (normalized) to a template MRI using the SEG-toolbox in SPM8 [39]. The new segmentation toolbox is an extension of the unified segmentation algorithm, which incorporates additional tissue priors for improved matching of the subject's MRI to the template [36], [37]. The AAL atlas was used to label the voxels in a subject's normalized co-registered MRI [40]. Subcortical structures were removed, and the voxels in the remaining 78 cortical regions of interest (ROIs) were used for further analysis [41], after inverse transformation to the patient's co-registered MRI.

Neuronal activity for the labeled voxels in the ROIs was reconstructed using a beamforming approach known as Synthetic Aperture Magnetometry (SAM) [42]. SAM works in a sequential fashion, where the activity for each voxel is reconstructed by selectively weighting the contribution from each MEG sensor to a voxel's time-series. This weighting is done such that the activity at a voxel is reconstructed without distortion, and at the same time the contribution from external (noise) sources is minimized [43], [44]. The beamformer weights are based on the covariance of the data and the forward solution (lead field) of a dipolar source at the voxel location, where data were band-pass filtered from 0.5–48 Hz. A time-window of, on average, 238 seconds (range: 164–282 sec.) was used for the computation of the data covariance matrix. We used broadband data for the estimation of the beamformer weights, as this avoids overestimation of covariance between channels [45]. The sensor-level data were subsequently projected through the beamformer weights, resulting in a time-series for each voxel. Each ROI contains many voxels and the number of voxels per ROI differed. In order to represent a ROI by a single time-series, we selected, for each ROI and frequency band separately, the voxel with maximum power in that frequency band [27]. The time-series for this voxel was used for further analysis, resulting in a total of 6 sets of 78 time-series (one for each frequency band, using six classic frequency bands: delta (0.5–4 Hz), theta (4–8 Hz), alpha1 (8–10 Hz), alpha2 (10–13Hz), beta (13–30 Hz), and gamma (30–48 Hz)). As in our previous studies we selected the first five artifact-free epochs of 4096 samples (6.555 seconds) from these time-series, based on careful visual inspection (PT) to obtain stable results [25], [46]–[53].

BrainWave software packing was used for this purpose and also for further analyses (version 0.9.58 available from http://home.kpn.nl/stam7883/brainwave.html).

### Functional Connectivity analysis

As a measure of FC, the phase lag index (PLI) was used [31], which calculates the asymmetry of the distribution of (instantaneous) phase differences between two time-series:
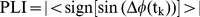
(1)Here the phase difference is defined in the interval [−π, π], <> denotes the mean value, sign stands for signum function and || indicates the absolute value. The PLI ranges between 0 (completely symmetric phase distribution) and 1 (completely asymmetric phase distribution). The rationale behind this approach is that field spread/volume conduction causes a zero phase lag between two time-series, and that a uniform distribution of phases occurs when there is no coupling at all. The presence of a consistent, non-zero, phase lag between two time-series therefore reflects true interactions that are unaffected by field spread/volume conduction or common sources [31]. Recent findings suggest that this method is capable of removing spurious coupling between ROIs at the expense of discarding any physiological interactions with zero lag [27].

The mean PLI over 5 epochs for every patient was computed for all ROIs, i.e. the full 78×78 adjacency matrix was estimated. All row elements in this adjacency matrix were averaged yielding 78 PLI values, one for each ROI. This is a measure of the overall connectivity of a region with all other regions (in the language of graph theory: weighted degree or node strength). Subsequently, these 78 mean PLI values were averaged in order to obtain one mean (whole brain) PLI for each subject. In addition, the mean PLI between ROIs within literature-based resting-state networks (RSN) was estimated for each subject. The rationale for this latter approach was that i) previous work [54], [55] has demonstrated that the integrity of RSNs is of importance for cognitive performance, and ii) one of our aims was to link altered functional connectivity in MS to cognitive performance. If A_ij_ is an element in the adjacency matrix for the RSN, then the mean PLI within a RSN that contains N ROIs is defined as:
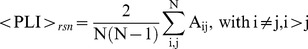
(2)Here i are row indices and j are column indices. Indices i and j denote the ROIs involved within the RSN of interest. Thus only PLI values in the adjacency matrix corresponding to connections between ROIs *within* a RSN were included for calculation of <PLI>_rsn_. The selection of RSNs and the ROIs selected for specific RSNs were based on a review paper [56]. For our analysis we included the default mode network (DMN), left and right fronto-parietal network (FPN), the executive function network, the sensorimotor network, the temporo-parietal network and the visual network (Table S1). We applied the rationale that connections within RSNs should be unique, and therefore made two modifications to the definition of the RSNs from the review [56]: the precuneus was substituted with the superior parietal gyrus in the FPN [57], and the inferior frontal gyrus was split into two parts, where inferior frontal gyrus pars operculis was assigned to the temporo-parietal network and inferior frontal gyrus pars triangularis was assigned to the FPN [56], [57].

### Statistical analysis

Firstly, mean whole brain PLI values were compared between groups with Mann-Whitney tests. Subsequently, as post-hoc analysis, mean PLI values per ROI were compared between groups by means of permutation analysis [58], where a null distribution for between-group differences (independent t-test) is derived by permuting group assignment and calculating a t-statistic after each permutation. If mean whole brain PLI values significantly differed between MS patients and healthy controls, as another post-hoc analyses, we used Mann-Whitney tests to determine whether there were differences in mean PLI values of RSNs (<PLI>_rsn_) between MS patients and healthy controls.

MRI atrophy parameters were compared between MS patients and healthy controls with independent t-tests and in absence of normality with the Mann-Whitney test. Normality was checked using histogram inspection and Kolmogorov-Smirnov tests of normality. Whenever the mean PLI or <PLI>_rsn_ was significantly different between MS patients and controls, correlations between these PLI values and cognition, EDSS, lesion load and MRI atrophy parameters were calculated using non parametric spearman's coefficients in the MS group and control group separately [59]. After correlation analyses and in case of normality, multiple regression analyses were used to determine if the association between volumes and PLI values were different between MS patients and controls. In these analyses volumes were regarded as effect modifiers and PLI values as outcome measures. These statistical analyses were performed using SPSS for windows v.18.

## Results

Characteristics of MS patients and healthy controls are listed in Table 1.

**Table 1:** Descriptive variables for controls and patients.

	Controls (N = 17)		Patients (N = 21)		p value
	Mean	± SD	Mean	± SD	
Age	39.8	±9.8	41.9	±7.7	0.49
Education(1–7)	5.9	±1.36	5.4	±1.33	0.52
Disease Duration			6.8	±0.9	–
NGMV (l)	0.84	±0.05	0.81	±0.04	0.037^*^
NWMV^a^ (l)	0.69	±0.03	0.66	±0.03	^–^
NBV (l)	1.53	±0.07	1.47	±0.05	0.006^*^
Total thalamic volume	0.021	±0.001	0.019	±0.002	0.004^*^
Cognition	0.04	±0.64	–0.19	±0.84	0.36
EDSS (1–10)^b^			2	(0–4.5)	–
T1 lesion load (mL)			1.05	±0.81	–
T2 lesion load (mL)			2.48	±2.03	–

NGMV, normalized gray matter volume; NWMV, normalized white matter volume; NBV, normalized brain volume; EDSS, expanded disability status scale.^a^ NWMV was not used for further analyses: lesion filling was not performed and therefore NWMV was not reliably estimated. ^b^ indicates median and range.* indicates significant differences between the two groups.

### MRI: Atrophy measures

NBV and NGMV were significantly lower in the patient group (independent t-tests t(36)  = −3.0, p = 0.006, t(36)  = −2.2, p = 0.037, respectively) (Table 1). Mann-Whitney test revealed significantly lower total thalamic volumes (U(36)  = 75.0, p = 0.004) in MS patients compared to healthy controls.

### Functional connectivity: ROI analysis

**Figure 1:**
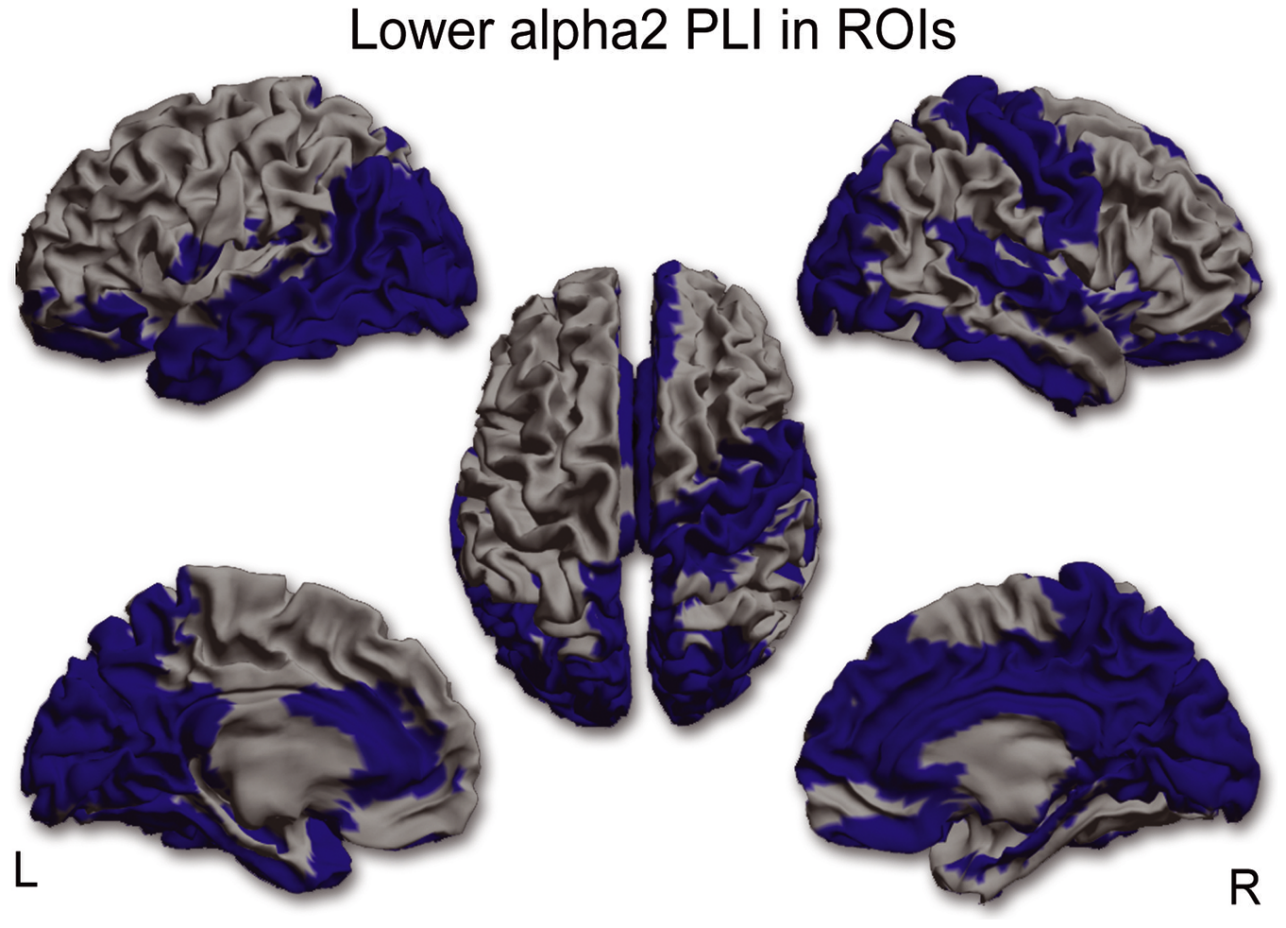
ROI analysis in the alpha2 band. In 40 ROIs corresponding PLI values in the alpha2 band were significantly lower in MS patients compared to healthy controls. This lower functional connectivity in the alpha2 band occurred in occipital, temporal, medial-parietal and medial frontal regions. (L = left hemisphere, R = right hemisphere).

**Figure 2:**
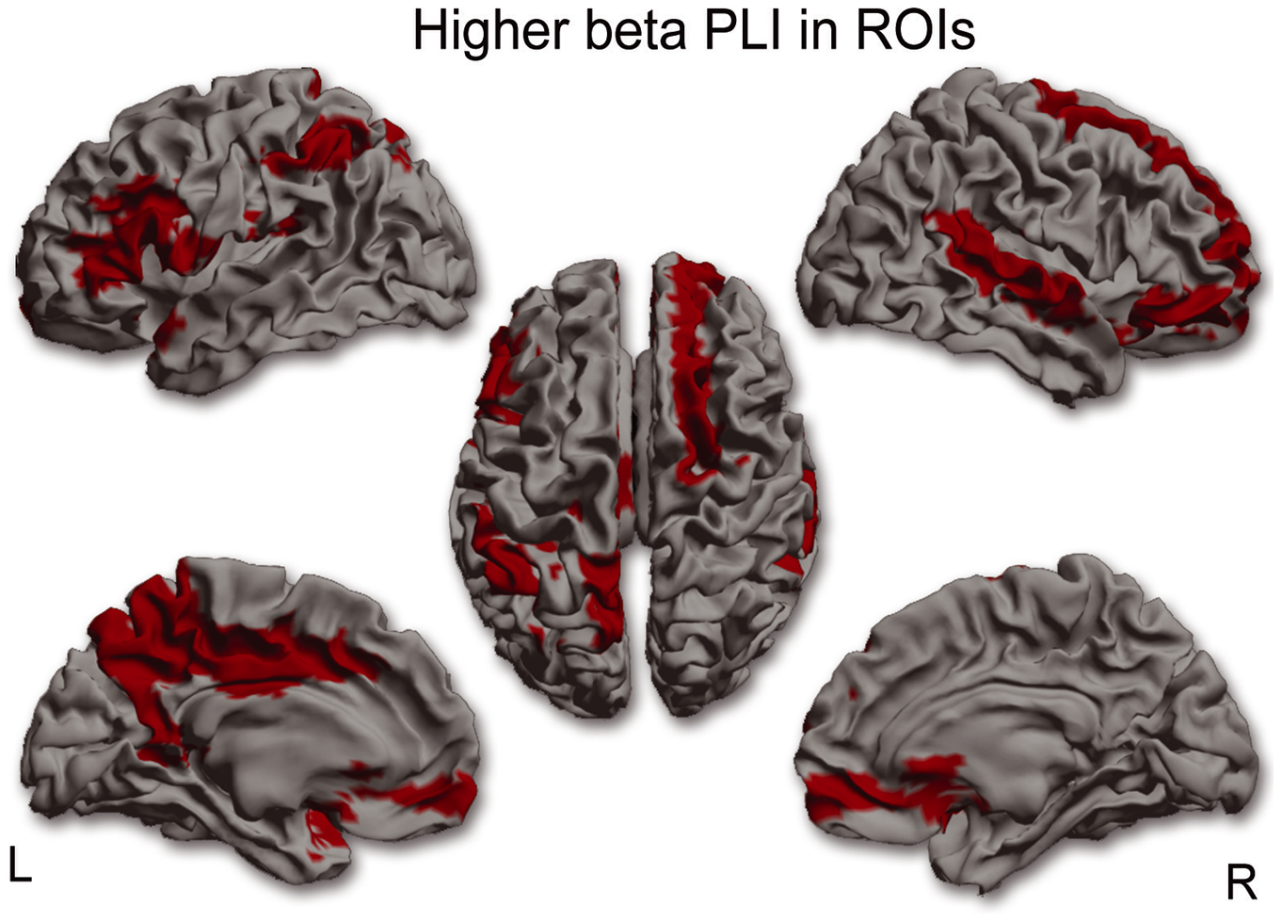
ROI analysis in the beta band. In 18 ROIs corresponding PLI values in the beta band were significantly higher in MS patients compared to healthy controls. This higher functional connectivity in the beta band occurred mainly in fronto-parietal and temporal regions. (L = left hemisphere, R = right hemisphere).

For each frequency band separately, mean (whole brain) PLI values were compared between MS patients and controls. Mean (whole brain) PLI was lower in the alpha2 (U(36)  = 89.0, p = 0.008) and higher in the beta band (U(36)  = 94.0, p = 0.012) in MS patients. Post-hoc analyses were performed in order to find out which ROIs were causing the global effect, which revealed significantly lower PLI values in 40 ROIs for the alpha2 band in MS patients (Figure 1 and see Table S2 for AAL regions), whereas in the beta band PLI values in 16 ROIS were significantly higher in MS patients (Figure 2 and see Table S2 for AAL regions).

Only mean (whole brain) beta PLI and EDSS correlated positively (r(19)  = 0.47, p = 0.031) in MS patients. There were no correlations between whole brain PLI and T1-, T2 lesion load or atrophy measures.

### Functional connectivity: resting-state networks

If mean (whole brain) PLI values differed in a specific frequency band between MS patients and healthy controls we calculated for each RSN the mean PLI within the network (<PLI>_rsn_) and compared these between MS patients and controls (see method section and [56] for definition of RSNs). Correlations with EDSS, cognition, T1-,T2 lesion load, thalamic volumes and global measures of atrophy were only computed when there were significant group differences for <PLI>_rsn_ in that frequency band.

**Figure 3:**
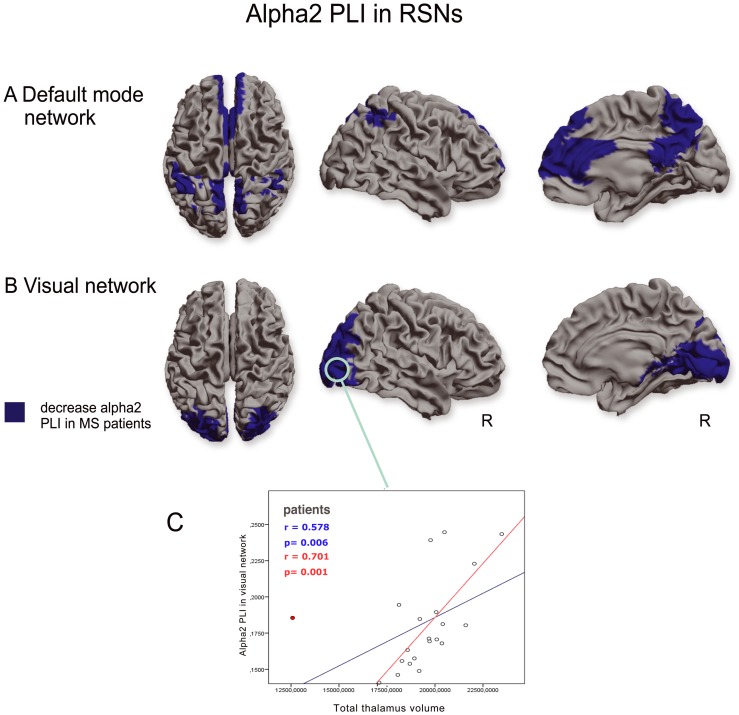
RSN analysis in the alpha2 band. Lower PLI values in the alpha2 band were found in the default mode network (a) and the visual network (b). Spearman correlation analysis in MS patients revealed a positive correlation between alpha2 PLI values in the visual network and thalamic volumes. (R = right hemisphere). Correlations are shown with (blue) and without (red) outlier (red dot).

In the alpha2 band two RSNs showed a significantly lower PLI value in MS patients: the DMN (U(36)  = 71.0, p = 0.001) (Figure 3a) and the visual network (U(36)  = 92 p = 0.01) (Figure 3b). The PLI value in the visual network in MS patients correlated positively with total thalamic volume (r(19)  = 0.58, p = 0.006) (Figure 3c and Table 2). After discarding outliers this relation remained significant (r(19)  = 0.7 p = 0.001). In the control group a positive, yet non-significant, correlation between total thalamic volume and PLI within the visual network was found as well.

**Table 2:** Correlations between functional connectivity within resting state networks and volumetric MRI measures in MS patients.

Spearman correlation coefficients								
	DMN alpha2		DMN beta		Temporo-parietal network beta		Visual network alpha2	
	R	p value	R	p value	R	p value	R	p value
NGMV	0.31	0.18	−0.13	0.58	−0.26	0.25	0.34	0.13
NBV	0.15	0.52	0.03	0.99	−0.024	0.92	0.42	0.059
Total thalamic volume	0.11	0.62	0.18	0.43	0.14	0.53	**0.58**	**0.006**

NGMV, normalized gray matter volume; NBV, normalized brain volume; NWMV, normalized white matter volume (NWMV was not used for further analyses since lesion filling had not performed and therefore NWMV was not reliably estimated); DMN alpha2, FC within the default mode network in the alpha2 band; DMN beta, FC within the default mode network in the beta band; temporo-parietal network beta, FC within the temporo-parietal network in the beta band; visual network alpha2, FC within the visual network in the alpha2 band.

**Figure 4:**
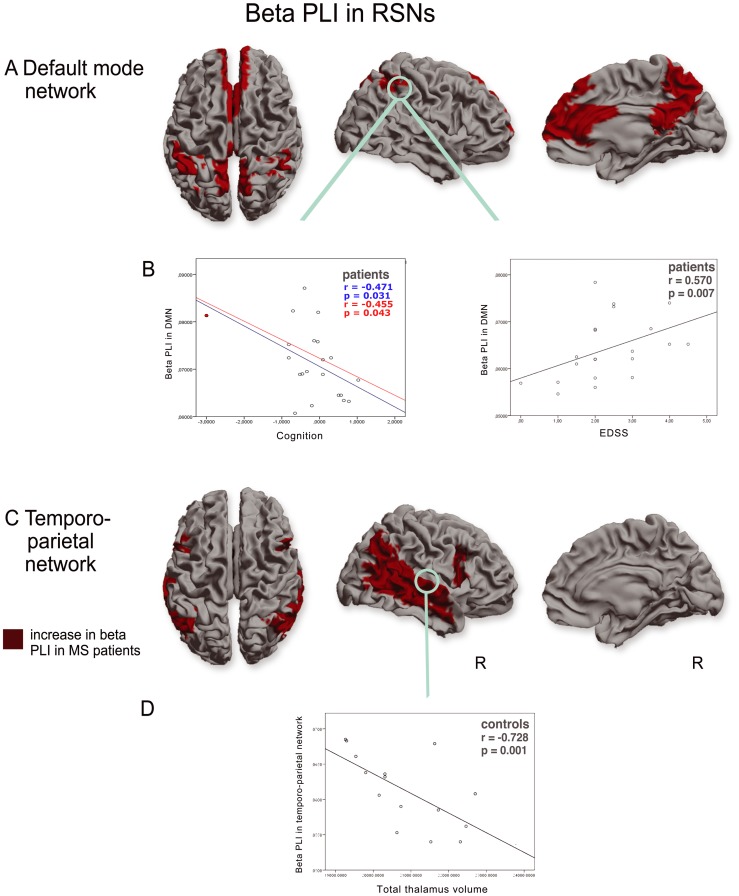
RSN analysis in the beta band. Higher PLI values in the beta band were found in the default mode network (a) and the temporoparietal network (c). Beta band PLI values in the default mode network correlated positively with physical disability (EDSS) and negatively with cognition in MS patients (b). Beta band PLI values in the temporoparietal network correlated negatively with thalamic volumes in healthy controls (d) but not in MS patients. (R = right hemisphere). Correlations are shown with (blue) and without (red) outlier (red dot).

Higher beta PLI values were found in the DMN (U(36)  = 93, p = 0.012) (Figure 4a) and the temporo-parietal network in MS patients (U(36)  = 106, p = 0.033) (Figure 4c). In the control group a negative correlation was found between beta band PLI in the temporo-parietal network and total thalamic volume (r(15)  = −0.73 p = 0.001) (Figure 4d), NGMV (r(15) = −0.52 p = 0.040), and NBV (r(15)  = −0.74 p = 0.001). In contrast, for the MS patient group, positive, yet non-significant, correlations were found between resting-state network beta band PLI and volumetric measures. Multiple regression analyses for the temporo-parietal network revealed that total thalamic volume had a significant different effect on beta PLI between MS patients and controls: Total thalamic volume in healthy controls was negatively associated with beta PLI within the temporo-parietal network (B  = −2.8×10^−6^, standardized β  = −0.7, p = 0.002) whereas total thalamic volume was positively, yet non significantly associated with beta PLI within the temporo-parietal network (B  = 1.5×10^−7^, standardized β  = 0.05, p = 0.8). No correlations were found between <PLI>_rsn_ of any RSN and T1-, T2 lesion load.

For every RSN for which there was a significant group difference in <PLI>_rsn_, correlations with EDSS and cognition were calculated. Significant correlations were only found in the DMN: beta PLI within this network correlated positively with EDSS (r(19)  = 0.57 p = 0.007) and showed a negative correlation with cognition (r(19)  = 0.47 p = 0.031) (Figure 4b). The correlation between cognition and beta PLI remained significant (r(19)  = −0.45 p = 0.043) after discarding outliers. There were no significant correlations between EDSS, age, education, cognition and T1-, T2 lesion load, NBV, or NWMV.

## Discussion

We used an atlas-based MEG beamformer approach to assess FC changes in early MS in relation to cognitive and clinical dysfunction, and in relation to MRI-based measures of structural integrity such as whole brain-, thalamic atrophy and lesion load. We observed the following changes: Firstly, whole brain FC analyses revealed lower FC in the alpha2 band and higher FC in the beta band in MS patients. Secondly, RSN analyses in MS patients revealed lower FC in the alpha2 band in two RSNs, the DMN and the visual processing network, and higher FC in the beta band in two RSNs, the DMN and the temporo-parietal network. Thirdly, changes in whole brain, and more specifically thalamic atrophy was related to altered FC in the visual network and the temporo-parietal network in respectively the alpha2 and beta band. Finally, most strikingly and clinically relevant was the observed correlation between higher beta band FC in the DMN and higher EDSS scores and poorer cognition.

Whole-brain FC was lower in the alpha2 and higher in the beta band for the MS patients. Both these rhythms are involved in top-down processing of rhythmic activity which is important for the integration of information in the cortex [60], [61]. Lower FC in the alpha2 band occurred in occipital, temporal, medial-parietal and medial-frontal regions. Higher FC in the beta band occurred mainly in fronto-parietal and temporal regions. In some regions, such as the right superior temporal gyrus and the precuneus, there was both higher FC in the beta band and lower FC in the alpha2 band. An explanation for this observation of a simultaneous change in different frequency bands could be that different sub-populations within a region (i.e. beyond the resolution of our approach) are involved in the generation of the different rhythms. Alternatively, we know from pharmacological MEG/EEG studies that increased inhibitory activity may lead to increased amplitude for beta oscillations and simultaneous decreased amplitude for alpha oscillations [62], [63]. Additionally, previous MEG/EEG studies in healthy controls revealed that cross-frequency phase synchrony between alpha and beta rhythms does appear in mainly parietal-occipital areas [64], [65]. Furthermore its has been suggested that cross-frequency phase synchrony could reflect coordination between different functional networks [64]. This is an important notion since our findings point out that the precuneus, an important hub region that is part of the DMN, displays both higher and lower FC in respectively the beta and alpha2 band. These interactions between frequency bands are therefore very interesting and possibly clinically important and warrant future exploration using cross-frequency phase synchrony analysis.

Previous MEG studies in early MS patients have also reported lower FC in the alpha [24] and alpha2 band [25], as well as higher FC in the beta band [25]. However, the latter study reported in addition higher FC in the theta and alpha1 band as well, using partially the same (but larger) patient group as used in the present study. The differences between this previous study and ours could possibly be explained by factors such as analysis level (sensor-space versus source-space), a different parcellation (sensor-groups versus atlas-based ROIs), a different FC metric (synchronization likelihood versus PLI), and therefore different sensitivity to spurious estimates of FC. An earlier EEG study in progressive MS patients found similarly a lower FC in the alpha band but also lower FC in the theta band [66]. As the study used progressive MS patients with higher disability scores and longer disease duration, it is difficult to compare these results. Indeed, it has been hypothesized that at different stages of the disease different patterns of functional reorganization occur [67]. Moreover, the aforementioned EEG study used coherence as a FC metric, which is sensitive to the amplitude of the timeseries [28], [66] and therefore to the effects of field spread/volume conduction.

In addition to group differences between whole brain average connectivity, we also found prominent differences for specific resting-state networks, and these differences correlated with structural measures and clinical and cognitive status. MS patients showed changes in the DMN, where, compared to the controls, both lower FC in the alpha2 band and higher FC in the beta band was seen. Both lower and higher FC in the DMN have been reported previously in an fMRI study in relatively late MS, with the anterior cingulate gyrus displaying lower FC, and the posterior cingulate gyrus showing lower FC in the core and higher FC at the periphery [16]. Another study that included relatively late MS patients found lower FC in the DMN [54]. Interestingly, in this latter study interactions between RSNs were analysed as well, which revealed lower FC between the DMN and the executive control network in MS patients. In our study, higher and lower FC in respectively the beta and alpha2 band in the DMN, as well as in other RSNs, could imply that these FC changes are linked between different RSNs, or between frequency bands within a single RSN. However interpreting this simultaneous higher and lower FC as a compensatory mechanism requires additional (longitudinal) data, and warrants caution at this time.

Apart from FC changes in the DMN, lower alpha2 FC in the visual network and higher beta FC in the temporo-parietal network were observed in MS patients. This is in partial agreement with recent fMRI studies that also reported lower FC in the visual network in MS [54], [68]. In our study altered FC in RSNs was only found in the alpha2 and beta band, which seems to be in line with results from a MEG study in healthy controls where they described that RSNs could be recovered from MEG recordings, especially in the alpha and beta band, and that these RSNs showed similar segregated topography as in fMRI [21].

An interesting finding in the present study is the relation between FC changes in RSNs and thalamic atrophy in MS, due to the ubiquitous connectivity of the thalamus with other regions throughout the brain. It has been reported previously that the thalamus is involved in many RSNs, including the DMN, sensorimotor network, visual network and the fronto-temporal network [69], [70]. Patients in the early phases of RRMS already show thalamic atrophy, as was also observed in this study. Importantly, we found correlations between thalamic atrophy and alpha2 band FC in the visual network in MS patients. Furthermore the effect of thalamic volume on beta FC in the temporo-parietal network was significantly different between MS patient and controls, again indicating an important role for the thalamus in relation to altered resting-state FC. Our observed association between thalamic atrophy and altered alpha band resting-state FC seems to be in agreement with the well-known role of the thalamus as co-generator of global alpha rhythms, and to a lesser extent of the beta rhythm in physiological conditions [67]–[71]. The exact neurophysiological and histopathological mechanisms that link altered FC and thalamic changes require further investigation, but could possibly relate to altered expression of cortico-thalamic metabotropic glutamate receptors in subcortical white matter in MS, as these are involved in generation of alpha rhythms [71], [72], a disbalance between inhibitory and excitatory neurons within the thalamus due to damaged interneurons in this structure in MS [73], alterations to parallel feedforward cortico-thalamo-cortical routes [74], or even vice versa: altered FC may lead to thalamic atrophy.

Two important clinically relevant findings from our study were the observed positive correlation between beta band FC in the DMN with physical disability (EDSS) and the negative correlation with cognition. Or, in other words, higher FC in the beta band is associated with a poorer clinical status, both regarding disability and cognition. It has been hypothesized that increases in beta band oscillations are associated with maintaining the present cognitive or sensorimotor status [75]. In the same way the authors predict that pathological enhancement of beta band synchrony is associated with a loss in flexibility of sensorimotor and cognitive processing. It has also been predicted that enhancement of beta band synchrony would predominantly occur in RSNs, as the default mode of the brain constitutes a state which seems to be distinguished by low expectation of ensuing change in the sensorimotor set [75]. Our findings seem to fit to both these predictions. A negative relationship between FC and cognition has also been found in an fMRI study [11], and these authors have suggested that a higher FC might be maladaptive and contribute to worsening of cognitive functions. In a computational study on Alzheimer's disease it has been shown that higher FC could reflect pathological disinhibition of cortical networks [76]. This mechanism could be a general common pathway in some stage of neurological diseases leading to higher FC. Taken together it seems questionable whether higher FC is always a compensatory mechanism to maintain cognitive abilities [67]. Model studies, using e.g. neural mass models, could help to clarify possible mechanisms of our observed increased FC in the beta band, combined with larger experimental and longitudinal patient studies.

When performing whole brain analyses, we only observed a weak correlation between mean beta PLI and EDSS, whereas stronger correlations were observed between beta PLI in the DMN and clinical parameters. Studying RSN FC with MEG seems therefore to have an advantage over whole-brain analyses. We expected to find a relation between FC in the sensorimotor network and physical disability, but this was not found. It is important to note that in this study there was no association between clinical parameters and volumetric MRI measures or lesion load. The observed relation between physical disability and FC within the DMN is difficult to interpret. Only one previous study has revealed a relationship between physical disability and altered FC in the DMN [54]. As the DMN seems to have such a central and vital role in the brain, it is considerable that damage to this network has an effect on many functions beyond cognition. If future studies are able to verify that physical disability is associated with higher FC in the beta band in the DMN, this may provide an objective assessment of clinical disability.

### Methodological considerations

RSN analyses of resting-state MEG source-space data is a relatively new approach. In our study regions attributed to each RSN were based on previous fMRI literature [56], [57] and RSNs we used are known to be highly robust, especially the DMN [56], [57]. Previous MEG beamforming studies using amplitude correlations revealed that MEG RSNs obtained after independent component analysis show strong overlap with fMRI obtained RSNs [21], [77], [78]. In contrast to these studies we used the PLI to quantify functional interactions between brain regions. The relation between amplitude correlation analyses (after application of an orthogonalisation step to remove spurious correlations) and phase synchronization methods has already been studied through simulations [78]. Importantly, the amplitude correlation between two signals was monotonically related to the magnitude of the phase difference between the carrier oscillations. This means that amplitude correlations could be less sensitive in picking up relations between two time series with small phase differences. In contrast, even when phase differences are small yet consistent, the PLI will capture such interactions. Moreover, PLI does not mix information about the magnitude of phase differences and the consistency of phase differences, enhancing the interpretability of observed interactions. Despite these differences the occurrences of spatial patterns obtained with both methods were largely overlapping. It remains an open, yet interesting, question how these different approaches relate in experimental data. Many more metrics of functional connectivity are available [79], yet the majority of these are sensitive to the effects of field spread and volume conduction. As mentioned above, novel approaches have been described to remove these biases before performing functional connectivity analyses [78], [80]. However, perhaps a more convenient approach is to use metrics that are inherently insensitive to these biases, such as the PLI or phase slope index [81].

The present study has some limitations. First of all, fMRI studies have previously revealed regional connectivity changes within the DMN in MS [16], [17]. In this first exploratory MEG study, such detailed regional differences were not studied yet. Secondly, the number of included subjects here was limited, possibly masking further MS-induced effects due to issues of statistical power. This also hampered the study of medication and gender effects. In addition, in such small datasets outliers could have a larger influence in the statistical outcome such as correlation or regression coefficients, although in the present study, removing outliers did not lead to significantly different results. Limited sample size was partially caused by exclusion of subjects due to sub-optimal MEG/MRI co-registration procedures and artefacts in the raw data. Future studies will use improved co-registration procedures [46], as well as a sophisticated artefact removal approach (temporal extension of Signal Space Separation (tSSS) [82], [83], in order to address these issues [37]. Thirdly, in this study RSN and regional FC were considered as post-hoc analyses after a main effect of disease had been found for a particular frequency band. Although it can be argued that such an explorative approach is justified, future studies are necessary to confirm our results, using an independent study sample in a hypothesis-driven study design that uses a more stringent statistical approach to the issue of multiple testing. Finally, the applied FC metric, PLI, is not influenced by power of the time series directly. However like any other FC metric, the PLI is influenced by the signal to noise ratio of the time series, and therefore indirectly depends on power [84]. Importantly though, the correlations between PLI and clinical parameters that we observed in this study, were not found for measures of relative power in the same data [85].

To conclude, changes in resting-state functional connectivity in MS were shown to be linked to neurodegenerative changes, as well as to clinical and cognitive status. This exploratory study could serve as a bridge to understand the effect of thalamic atrophy on clinical and cognitive status through altered functional connectivity, although future longitudinal studies are required. Moreover, the assessment of clinical and cognitive status is difficult, but our results suggest that the frequency-specific functional connectivity within MEG resting-state networks could be used as new imaging-based biomarkers, which future studies using other cohorts now need to validate.

## Supporting Information

Table S1 Literature based RSNs: ROIs that were included in each resting-state network are listed. All RSNs consist of a unique selection of connections between ROIs in the AAL atlas.
(DOC)

Table S2 Regions of interest involved in functional connectivity changes.
(DOC)

## References

[1] Barkhof F (2002) The clinico-radiological paradox in multiple sclerosis revisited. Curr Opin Neurol 15: 239–245. 12045719 10.1097/00019052-200206000-00003

[2] Filippi M, Rocca MA, Barkhof F, Bruck W, Chen JT, et al (2012) Association between pathological and MRI findings in multiple sclerosis. Lancet Neurol 11: 349–360. 22441196 10.1016/S1474-4422(12)70003-0

[3] Audoin B, Zaaraoui W, Reuter F, Rico A, Malikova I, et al (2010) Atrophy mainly affects the limbic system and the deep grey matter at the first stage of multiple sclerosis. J Neurol Neurosurg Psychiatry 81: 690–695. 20392976 10.1136/jnnp.2009.188748

[4] Tao G, Datta S, He R, Nelson F, Wolinsky JS, et al (2009) Deep gray matter atrophy in multiple sclerosis: a tensor based morphometry. J Neurol Sci 282: 39–46. 19168189 10.1016/j.jns.2008.12.035 PMC2744867

[5] Houtchens MK, Benedict RH, Killiany R, Sharma J, Jaisani Z, et al (2007) Thalamic atrophy and cognition in multiple sclerosis. Neurology 69: 1213–1223. 17875909 10.1212/01.wnl.0000276992.17011.b5

[6] Batista S, Zivadinov R, Hoogs M, Bergsland N, Heininen-Brown M, et al (2012) Basal ganglia, thalamus and neocortical atrophy predicting slowed cognitive processing in multiple sclerosis. J Neurol 259: 139–146. 21720932 10.1007/s00415-011-6147-1

[7] Schoonheim MM, Popescu V, Rueda Lopes FC, Wiebenga OT, Vrenken H, et al (2012) Subcortical atrophy and cognition: sex effects in multiple sclerosis. Neurology 79: 1754–1761. 23019265 10.1212/WNL.0b013e3182703f46

[8] Cader S, Cifelli A, Abu-Omar Y, Palace J, Matthews PM (2006) Reduced brain functional reserve and altered functional connectivity in patients with multiple sclerosis. Brain 129: 527–537. 16251214 10.1093/brain/awh670

[9] Leavitt VM, Wylie G, Genova HM, Chiaravalloti ND, DeLuca J (2012) Altered effective connectivity during performance of an information processing speed task in multiple sclerosis. Mult Scler 18: 409–417. 21965419 10.1177/1352458511423651

[10] Parisi L, Rocca MA, Valsasina P, Panicari L, Mattioli F, et al.. (2012) Cognitive rehabilitation correlates with the functional connectivity of the anterior cingulate cortex in patients with multiple sclerosis. Brain Imaging Behav. 10.1007/s11682-012-9160-9 22528829

[11] Hawellek DJ, Hipp JF, Lewis CM, Corbetta M, Engel AK (2011) Increased functional connectivity indicates the severity of cognitive impairment in multiple sclerosis. Proc Natl Acad Sci U S A 108: 19066–19071. 22065778 10.1073/pnas.1110024108 PMC3223469

[12] Helekar SA, Shin JC, Mattson BJ, Bartley K, Stosic M, et al (2010) Functional brain network changes associated with maintenance of cognitive function in multiple sclerosis. Front Hum Neurosci 4: 219. 21152340 10.3389/fnhum.2010.00219 PMC2996259

[13] Damoiseaux JS, Rombouts SA, Barkhof F, Scheltens P, Stam CJ, et al (2006) Consistent resting-state networks across healthy subjects. Proc Natl Acad Sci U S A 103: 13848–13853. 16945915 10.1073/pnas.0601417103 PMC1564249

[14] Broyd SJ, Demanuele C, Debener S, Helps SK, James CJ, et al (2009) Default-mode brain dysfunction in mental disorders: a systematic review. Neurosci Biobehav Rev 33: 279–296. 18824195 10.1016/j.neubiorev.2008.09.002

[15] Hafkemeijer A, van der Grond J, Rombouts SA (2012) Imaging the default mode network in aging and dementia. Biochim Biophys Acta 1822: 431–441. 21807094 10.1016/j.bbadis.2011.07.008

[16] Bonavita S, Gallo A, Sacco R, Corte MD, Bisecco A, et al (2011) Distributed changes in default-mode resting-state connectivity in multiple sclerosis. Mult Scler 17: 411–422. 21239414 10.1177/1352458510394609

[17] Rocca MA, Valsasina P, Absinta M, Riccitelli G, Rodegher ME, et al (2010) Default-mode network dysfunction and cognitive impairment in progressive MS. Neurology 74: 1252–1259. 20404306 10.1212/WNL.0b013e3181d9ed91

[18] Faivre A, Rico A, Zaaraoui W, Crespy L, Reuter F, et al (2012) Assessing brain connectivity at rest is clinically relevant in early multiple sclerosis. Mult Scler 18: 1251–1258. 22307385 10.1177/1352458511435930

[19] Logothetis NK (2008) What we can do and what we cannot do with fMRI. Nature 453: 869–878. 18548064 10.1038/nature06976

[20] Singh KD (2012) Which “neural activity” do you mean? fMRI, MEG, oscillations and neurotransmitters. Neuroimage 62: 1121–1130. 22248578 10.1016/j.neuroimage.2012.01.028

[21] de Pasquale F, Della PS, Snyder AZ, Marzetti L, Pizzella V, et al (2012) A cortical core for dynamic integration of functional networks in the resting human brain. Neuron 74: 753–764. 22632732 10.1016/j.neuron.2012.03.031 PMC3361697

[22] Dell'Acqua ML, Landi D, Zito G, Zappasodi F, Lupoi D, et al (2010) Thalamocortical sensorimotor circuit in multiple sclerosis: an integrated structural and electrophysiological assessment. Hum Brain Mapp 31: 1588–1600. 20162580 10.1002/hbm.20961 PMC6871076

[23] Tecchio F, Zito G, Zappasodi F, Dell' Acqua ML, Landi D, et al (2008) Intra-cortical connectivity in multiple sclerosis: a neurophysiological approach. Brain 131: 1783–1792. 18502782 10.1093/brain/awn087

[24] Cover KS, Vrenken H, Geurts JJ, van Oosten BW, Jelles B, et al (2006) Multiple sclerosis patients show a highly significant decrease in alpha band interhemispheric synchronization measured using MEG. Neuroimage 29: 783–788. 16226894 10.1016/j.neuroimage.2005.08.048

[25] Schoonheim MM, Geurts JJ, Landi D, Douw L, van der Meer ML, et al.. (2011) Functional connectivity changes in multiple sclerosis patients: A graph analytical study of MEG resting state data. Hum Brain Mapp. 10.1002/hbm.21424 PMC6870283 21954106

[26] Hardmeier M, Schoonheim MM, Geurts JJ, Hillebrand A, Polman CH, et al (2012) Cognitive dysfunction in early multiple sclerosis: altered centrality derived from resting-state functional connectivity using magneto-encephalography. PLoS One 7: e42087. 22848712 10.1371/journal.pone.0042087 PMC3407108

[27] Hillebrand A, Barnes GR, Bosboom JL, Berendse HW, Stam CJ (2012) Frequency-dependent functional connectivity within resting-state networks: an atlas-based MEG beamformer solution. Neuroimage 59: 3909–3921. 22122866 10.1016/j.neuroimage.2011.11.005 PMC3382730

[28] Peraza LR, Asghar AU, Green G, Halliday DM (2012) Volume conduction effects in brain network inference from electroencephalographic recordings using phase lag index. J Neurosci Methods 207: 189–199. 22546477 10.1016/j.jneumeth.2012.04.007

[29] David O, Garnero L, Cosmelli D, Varela FJ (2002) Estimation of neural dynamics from MEG/EEG cortical current density maps: application to the reconstruction of large-scale cortical synchrony. IEEE Trans Biomed Eng 49: 975–987. 12214887 10.1109/TBME.2002.802013

[30] Hui HB, Pantazis D, Bressler SL, Leahy RM (2010) Identifying true cortical interactions in MEG using the nulling beamformer. Neuroimage 49: 3161–3174. 19896541 10.1016/j.neuroimage.2009.10.078 PMC2818446

[31] Stam CJ, Nolte G, Daffertshofer A (2007) Phase lag index: assessment of functional connectivity from multi channel EEG and MEG with diminished bias from common sources. Hum Brain Mapp 28: 1178–1193. 17266107 10.1002/hbm.20346 PMC6871367

[32] Polman CH, Reingold SC, Edan G, Filippi M, Hartung HP, et al (2005) Diagnostic criteria for multiple sclerosis: 2005 revisions to the “McDonald Criteria”. Ann Neurol 58: 840–846. 16283615 10.1002/ana.20703

[33] Lublin FD, Reingold SC (1996) Defining the clinical course of multiple sclerosis: results of an international survey. National Multiple Sclerosis Society (USA) Advisory Committee on Clinical Trials of New Agents in Multiple Sclerosis. Neurology 46: 907–911. 8780061 10.1212/wnl.46.4.907

[34] Kurtzke JF (1983) Rating neurologic impairment in multiple sclerosis: an expanded disability status scale (EDSS). Neurology 33: 1444–1452. 6685237 10.1212/wnl.33.11.1444

[35] Smith SM, Zhang Y, Jenkinson M, Chen J, Matthews PM, et al (2002) Accurate, robust, and automated longitudinal and cross-sectional brain change analysis. Neuroimage 17: 479–489. 12482100 10.1006/nimg.2002.1040

[36] Vrba J AGBK. (1999) 151-Channel whole-cortex MEG system for seated or supine positions. Recent Advances in Biomagnetism Sendai, Japan: Tohoku University Press.

[37] Gross J, Baillet S, Barnes GR, Henson RN, Hillebrand A, et al (2012) Good practice for conducting and reporting MEG research. Neuroimage 65C: 349–363. 10.1016/j.neuroimage.2012.10.001 PMC3925794 23046981

[38] Brookes MJ, Vrba J, Robinson SE, Stevenson CM, Peters AM, et al (2008) Optimising experimental design for MEG beamformer imaging. Neuroimage 39: 1788–1802. 18155612 10.1016/j.neuroimage.2007.09.050

[39] Friston KJ, Holmes P, Worsley KJ, Poline JP, Frith CD, et al (2004) Statistical parametric maps in functional imaging: A general linear approach. Human Brain Mapping 2: 189–210.

[40] Tzourio-Mazoyer N, Landeau B, Papathanassiou D, Crivello F, Etard O, et al (2002) Automated anatomical labeling of activations in SPM using a macroscopic anatomical parcellation of the MNI MRI single-subject brain. Neuroimage 15: 273–289. 11771995 10.1006/nimg.2001.0978

[41] Gong G, He Y, Concha L, Lebel C, Gross DW, et al (2009) Mapping anatomical connectivity patterns of human cerebral cortex using in vivo diffusion tensor imaging tractography. Cereb Cortex 19: 524–536. 18567609 10.1093/cercor/bhn102 PMC2722790

[42] Robinson SE, Vrba J. (1999) Functional neuroimaging by synthetic aperture magnetometry. In: Yoshimoto M, Kotani S, Kuriki H, Karibe N, Nakatato E, editors. Recent advances in biomagnetism. Tohoku University Press, Sendai. 302–305.

[43] Hillebrand A, Barnes GR (2005) Beamformer analysis of MEG data. Int Rev Neurobiol 68: 149–171. 16443013 10.1016/S0074-7742(05)68006-3

[44] Hillebrand A, Singh KD, Holliday IE, Furlong PL, Barnes GR (2005) A new approach to neuroimaging with magnetoencephalography. Hum Brain Mapp 25: 199–211. 15846771 10.1002/hbm.20102 PMC6871673

[45] Barnes GR, Hillebrand A (2003) Statistical flattening of MEG beamformer images. Hum Brain Mapp 18: 1–12. 12454907 10.1002/hbm.10072 PMC6871854

[46] van Dellen E, de Witt Hamer PC, Douw L, Klein K, Heimans JJ, et al (2013) Connectivity in MEG resting-state networks increases after resective surgery for low-grade glioma and correlates with improved cognitive performance. Neuroimage:clinical 2: 1–7. 10.1016/j.nicl.2012.10.007 PMC3777771 24179752

[47] Bartolomei F, Bosma I, Klein M, Baayen JC, Reijneveld JC, et al (2006) Disturbed functional connectivity in brain tumour patients: evaluation by graph analysis of synchronization matrices. Clin Neurophysiol 117: 2039–2049. 16859985 10.1016/j.clinph.2006.05.018

[48] Bosma I, Stam CJ, Douw L, Bartolomei F, Heimans JJ, et al (2008) The influence of low-grade glioma on resting state oscillatory brain activity: a magnetoencephalography study. J Neurooncol 88: 77–85. 18259691 10.1007/s11060-008-9535-3 PMC2295256

[49] Douw L, Baayen H, Bosma I, Klein M, Vandertop P, et al (2008) Treatment-related changes in functional connectivity in brain tumor patients: a magnetoencephalography study. Exp Neurol 212: 285–290. 18534578 10.1016/j.expneurol.2008.03.013

[50] Douw L, van DE, Baayen JC, Klein M, Velis DN, et al (2010) The lesioned brain: still a small-world? Front Hum Neurosci 4: 174. 21120140 10.3389/fnhum.2010.00174 PMC2991225

[51] Douw L, Baayen JC, Klein M, Velis D, Alpherts WC, et al (2009) Functional connectivity in the brain before and during intra-arterial amobarbital injection (Wada test). Neuroimage 46: 584–588. 19269336 10.1016/j.neuroimage.2009.02.034

[52] Stam CJ, Jones BF, Manshanden I, van Cappellen van Walsum AM, Montez T, et al (2006) Magnetoencephalographic evaluation of resting-state functional connectivity in Alzheimer's disease. Neuroimage 32: 1335–1344. 16815039 10.1016/j.neuroimage.2006.05.033

[53] Stam CJ, de HW, Daffertshofer A, Jones BF, Manshanden I, et al (2009) Graph theoretical analysis of magnetoencephalographic functional connectivity in Alzheimer's disease. Brain 132: 213–224. 18952674 10.1093/brain/awn262

[54] Rocca MA, Valsasina P, Martinelli V, Misci P, Falini A, et al (2012) Large-scale neuronal network dysfunction in relapsing-remitting multiple sclerosis. Neurology 79: 1449–1457. 22955126 10.1212/WNL.0b013e31826d5f10

[55] Bonavita S, Gallo A, Sacco R, Corte MD, Bisecco A, et al (2011) Distributed changes in default-mode resting-state connectivity in multiple sclerosis. Mult Scler 17: 411–422. 21239414 10.1177/1352458510394609

[56] Rosazza C, Minati L (2011) Resting-state brain networks: literature review and clinical applications. Neurol Sci 32: 773–785. 21667095 10.1007/s10072-011-0636-y

[57] van den Heuvel MP, Hulshoff Pol HE (2010) Exploring the brain network: a review on resting-state fMRI functional connectivity. Eur Neuropsychopharmacol 20: 519–534. 20471808 10.1016/j.euroneuro.2010.03.008

[58] Nichols TE, Holmes AP (2002) Nonparametric permutation tests for functional neuroimaging: a primer with examples. Hum Brain Mapp 15: 1–25. 11747097 10.1002/hbm.1058 PMC6871862

[59] Rousselet GA, Pernet CR (2012) Improving standards in brain-behavior correlation analyses. Front Hum Neurosci 6: 119. 22563313 10.3389/fnhum.2012.00119 PMC3342588

[60] Engel AK, Fries P, Singer W (2001) Dynamic predictions: oscillations and synchrony in top-down processing. Nat Rev Neurosci 2: 704–716. 11584308 10.1038/35094565

[61] Palva S, Palva JM (2007) New vistas for alpha-frequency band oscillations. Trends Neurosci 30: 150–158. 17307258 10.1016/j.tins.2007.02.001

[62] Jensen O, Goel P, Kopell N, Pohja M, Hari R, et al (2005) On the human sensorimotor-cortex beta rhythm: sources and modeling. Neuroimage 26: 347–355. 15907295 10.1016/j.neuroimage.2005.02.008

[63] Liley DT, Cadusch PJ, Gray M, Nathan PJ (2003) Drug-induced modification of the system properties associated with spontaneous human electroencephalographic activity. Phys Rev E Stat Nonlin Soft Matter Phys 68: 051906. 14682819 10.1103/PhysRevE.68.051906

[64] Nikulin VV, Brismar T (2006) Phase synchronization between alpha and beta oscillations in the human electroencephalogram. Neuroscience 137: 647–657. 16338092 10.1016/j.neuroscience.2005.10.031

[65] Palva JM, Palva S, Kaila K (2005) Phase synchrony among neuronal oscillations in the human cortex. J Neurosci 25: 3962–3972. 15829648 10.1523/JNEUROSCI.4250-04.2005 PMC6724920

[66] Leocani L, Locatelli T, Martinelli V, Rovaris M, Falautano M, et al (2000) Electroencephalographic coherence analysis in multiple sclerosis: correlation with clinical, neuropsychological, and MRI findings. J Neurol Neurosurg Psychiatry 69: 192–198. 10896692 10.1136/jnnp.69.2.192 PMC1737052

[67] Schoonheim MM, Geurts JJ, Barkhof F (2010) The limits of functional reorganization in multiple sclerosis. Neurology 74: 1246–1247. 20404304 10.1212/WNL.0b013e3181db9957

[68] Gallo A, Esposito F, Sacco R, Docimo R, Bisecco A, et al (2012) Visual resting-state network in relapsing-remitting MS with and without previous optic neuritis. Neurology 79: 1458–1465. 22972637 10.1212/WNL.0b013e31826d5eea

[69] De Luca M, Beckmann CF, De SN, Matthews PM, Smith SM (2006) fMRI resting state networks define distinct modes of long-distance interactions in the human brain. Neuroimage 29: 1359–1367. 16260155 10.1016/j.neuroimage.2005.08.035

[70] Mantini D, Perrucci MG, Del GC, Romani GL, Corbetta M (2007) Electrophysiological signatures of resting state networks in the human brain. Proc Natl Acad Sci U S A 104: 13170–13175. 17670949 10.1073/pnas.0700668104 PMC1941820

[71] Hughes SW, Crunelli V (2005) Thalamic mechanisms of EEG alpha rhythms and their pathological implications. Neuroscientist 11: 357–372. 16061522 10.1177/1073858405277450

[72] Geurts JJ, Wolswijk G, Bo L, van d, V, Polman CH, et al (2003) Altered expression patterns of group I and II metabotropic glutamate receptors in multiple sclerosis. Brain 126: 1755–1766. 12805104 10.1093/brain/awg179

[73] Bo L (2009) The histopathology of grey matter demyelination in multiple sclerosis. Acta Neurol Scand 120: 51–57. 10.1111/j.1600-0404.2009.01216.x 19566500

[74] Sherman SM (2007) The thalamus is more than just a relay. Curr Opin Neurobiol 17: 417–422. 17707635 10.1016/j.conb.2007.07.003 PMC2753250

[75] Engel AK, Fries P (2010) Beta-band oscillations–signalling the status quo? Curr Opin Neurobiol 20: 156–165. 20359884 10.1016/j.conb.2010.02.015

[76] de Haan W, Mott K, van Straaten EC, Scheltens P, Stam CJ (2012) Activity dependent degeneration explains hub vulnerability in Alzheimer's disease. PLoS Comput Biol 8: e1002582. 22915996 10.1371/journal.pcbi.1002582 PMC3420961

[77] Brookes MJ, Woolrich M, Luckhoo H, Price D, Hale JR, et al (2011) Investigating the electrophysiological basis of resting state networks using magnetoencephalography. Proc Natl Acad Sci U S A 108: 16783–16788. 21930901 10.1073/pnas.1112685108 PMC3189080

[78] Hipp JF, Hawellek DJ, Corbetta M, Siegel M, Engel AK. (2012) Large-scale cortical correlation structure of spontaneous oscillatory activity. Nat Neurosci. 10.1038/nn.3101 PMC3861400 22561454

[79] Pereda E, Quiroga RQ, Bhattacharya J (2005) Nonlinear multivariate analysis of neurophysiological signals. Prog Neurobiol 77: 1–37. 16289760 10.1016/j.pneurobio.2005.10.003

[80] Brookes MJ, Woolrich MW, Barnes GR (2012) Measuring functional connectivity in MEG: a multivariate approach insensitive to linear source leakage. NeuroImage 63: 910–920. 22484306 10.1016/j.neuroimage.2012.03.048 PMC3459100

[81] Nolte G, Ziehe A, Nikulin VV, Schlogl A, Kramer N, et al (2008) Robustly estimating the flow direction of information in complex physical systems. Phys Rev Lett 100: 234101. 18643502 10.1103/PhysRevLett.100.234101

[82] Taulu S, Simola J (2006) Spatiotemporal signal space separation method for rejecting nearby interference in MEG measurements. Phys Med Biol 51: 1759–1768. 16552102 10.1088/0031-9155/51/7/008

[83] Taulu S, Hari R (2009) Removal of magnetoencephalographic artifacts with temporal signal-space separation: demonstration with single-trial auditory-evoked responses. Hum Brain Mapp 30: 1524–1534. 18661502 10.1002/hbm.20627 PMC6871056

[84] Muthukumaraswamy SD, Singh KD (2011) A cautionary note on the interpretation of phase-locking estimates with concurrent changes in power. Clin Neurophysiol 122: 2324–2325. 21543253 10.1016/j.clinph.2011.04.003

[85] Meer vdM, Tewarie P, Schoonheim M, Douw L, Barkhof F, et al.. (2012) Clinical disability in MS correlates with resting-state oscillatory brain activity: an MEG source-space study. in preparation. 10.1016/j.nicl.2013.05.003 PMC3777767 24179824

