# Supplementary material for: Cognitive and Clinical Dysfunction, Altered MEG Resting-State Networks and Thalamic Atrophy in Multiple Sclerosis
Source: PLoS One. 2013 Jul 31;8(7):e69318. doi: 10.1371/journal.pone.0069318 (PMC3729968; doi:10.1371/journal.pone.0069318)
Supplement: Table S1 — Literature based RSNs: ROIs that were included in each resting-state network are listed. All RSNs consist of a unique selection of connections between ROIs in the AAL atlas. (DOC) [file pone.0069318.s001.doc]

Table S1: Literature based RSNs: ROIs that were included in each resting-state network are listed. All RSNs consist of a unique selection of connections between ROIs in the AAL atlas.

| **Resting-state networks** | Selection of ROIs in the AAL atlas |
| --- | --- |
| Auditory component | Superior temporal gyrus, heschl’s gyrus, insula, postcentral gyrus |
| Default mode | Precuneus, posterior en anterior cingulate, inferior parietal gyrus, mesial prefrontal gyrus |
| Executive control | Medial frontal cortex, superior frontal gyrus, anterior cingulate |
| Frontoparietal (left/right) | inferior frontal gyrus pars triangularis, medial frontal gyrus, inferior parietal gyrus, superior parietal gyrus, angular gyrus |
| Sensorimotor | Precentral gyrus, postcentral gyrus, supplementary motor area |
| Temporo-parietal component | Inferior frontal gyrus pars operculis, medial temporal gyrus, superior temporal gyrus, angular gyrus |
| Visual component | Occipital_superior gyrus, Occipital_middle gyrus, Occipital_inferior gyrus, Calcarine gyrus, Lingual gyrus |
