# Supplementary material for: Cognitive and Clinical Dysfunction, Altered MEG Resting-State Networks and Thalamic Atrophy in Multiple Sclerosis
Source: PLoS One. 2013 Jul 31;8(7):e69318. doi: 10.1371/journal.pone.0069318 (PMC3729968; doi:10.1371/journal.pone.0069318)
Supplement: Table S2 — Regions of interest involved in functional connectivity changes. (DOC) [file pone.0069318.s002.doc]

| Table S2: Regions of interest involved in functional connectivity changes | | | |
| --- | --- | --- | --- |
| Lower alpha2 PLI in AAL regions | | Higher beta PLI in AAL regions | |
| AAL region | T-value | AAL region | T-value |
| Olfactory_L | -2.6774 | Olfactory_L | 2.2336 |
| Frontal_Sup_Orb_L | -2.1146 | Frontal_Med_Orb_L | 2.1529 |
| Frontal_Inf_Orb_L | -2.2027 | Frontal_Inf_Oper_L | 2.0662 |
| Rolandic_Oper_L | -2.2.398 | Frontal_Inf_Tri_L | 2.2361 |
| Angular_L | -2.2.125 | Rolandic_Oper_L | 2.5095 |
| Precuneus_L | -2.9.361 | Parietal_Inf_L | 2.3601 |
| Occipital_Sup_L | -2.4559 | Precuneus_L | 2.0316 |
| Occipital_Mid_L | -3.1707 | Temporal_Pole_Sup_L | 2.7120 |
| Occipital_Inf_L | -2.4853 | ParaHippocampal_L | 2.0580 |
| Calcarine_L | -1.9677 | Cingulum_Mid_L | 2.2675 |
| Cuneus_L | -2.2252 | Olfactory_R | 2.1059 |
| Lingual_L | -2.8002 | Frontal_Med_Orb_R | 2.8213 |
| Fusiform_L | -3.2080 | Frontal_Mid_Orb_R | 3.1585 |
| Temporal_Mid_L | -2.1673 | Frontal_Inf_Orb_R | 2.4659 |
| Temporal_Inf_L | -1.9901 | Frontal_Sup_R | 2.1315 |
| Temporal_Pole_Sup_L | -2.8085 | Temporal_Sup_R | 3.2478 |
| Temporal_Pole_Mid_L | -3.6363 |  |  |
| Cingulum_Ant_L | -3.2009 |  |  |
| Cingulum_Post_L | -1.9896 |  |  |
| Rectus_R | -2.1523 |  |  |
| Olfactory_R | -2.0510 |  |  |
| Frontal_Sup_Orb_R | -2.2997 |  |  |
| Frontal_Sup_Medial_R | -2.2092 |  |  |
| Paracentral_Lobule_R | -2.0748 |  |  |
| Precentral_R | -2.3587 |  |  |
| Postcentral_R | -2.4734 |  |  |
| Precuneus_R | -3.7711 |  |  |
| Occipital_Sup_R | -2.2529 |  |  |
| Occipital_Mid_R | -1.9866 |  |  |
| Occipital_Inf_R | -2.1812 |  |  |
| Calcarine_R | -3.1729 |  |  |
| Cuneus_R | -3.9377 |  |  |
| Lingual_R | -2.3646 |  |  |
| Temporal_Sup_R | -2.4536 |  |  |
| Temporal_Inf_R | -2.4031 |  |  |
| ParaHippocampal_R | -2.1725 |  |  |
| Cingulum_Ant_R | -2.4849 |  |  |
| Cingulum_Mid_R | -2.5664 |  |  |
| Cingulum_Post_R | -2.6684 |  |  |
| Insula_R | -2.1395 |  |  |

In 40 ROIs, PLI values in the alpha2 band were significantly lower (p<0.05) in MS patients compared to controls, and in 16 ROIs in the beta band the values were significantly higher (p<0.05). PLI values mentioned here are the average of all PLI values between a ROI and all other ROIs in the network.
